# Executive Functions Training Improves Language Abilities in Aphasia Rehabilitation: A Systematic Review

**DOI:** 10.3390/jpm15030092

**Published:** 2025-02-27

**Authors:** Laura Culicetto, Desirèe Latella, Viviana Lo Buono, Fabio Orecchio, Anna Maria Murdaca, Angelo Quartarone, Silvia Marino

**Affiliations:** 1 IRCCS Centro Neurolesi “Bonino-Pulejo”, S.S. 113 Via Palermo C. da Casazza, 98124 Messina, Italy; laura.culicetto@irccsme.it (L.C.); viviana.lobuono@irccsme.it (V.L.B.); angelo.quartarone@irccsme.it (A.Q.); silvia.marino@irccsme.it (S.M.); 2 Faculty of Human Sciences, Università Telematica Pegaso, 80143 Naples, Italy; fabio.orecchio@unipegaso.it; 3 Dipartimento di Studi Classici, Linguistici e Della Formazione, Università degli Studi di Enna “Kore”, 94100 Enna, Italy; annamaria.murdaca@unikore.it

**Keywords:** aphasia treatment, language skill rehabilitation, language recovery, working memory training, non-invasive neuromodulation

## Abstract

**Background/Objectives**: In recent years, the popularity of non-verbal cognitive training for aphasia has increased. Building on evidence that language abilities engage brain areas involved in executive functions (EFs) processing, this review aims to analyze the utility of EFs training alone or combined with traditional rehabilitation approaches to improve language abilities in aphasia. **Methods**: Systematic searches were performed in four databases evaluating studies focusing on the effects of EFs training in language rehabilitation, yielding 185 studies. After reading the full text of the selected studies and applying predefined inclusion criteria, nine studies were included based on pertinence and relevance to the topic. This systematic review has been registered in the Prospective Register of Systematic Reviews (PROSPERO 2024) with the number CRD42024519087. **Results**: The results of the analyzed studies indicate that various EFs training methods, such as computer-assisted executive control training, Cognitive Flexibility in Aphasia Therapy (CFAT), and the Dr. Neuronowski^®^ program, as well as the combination of transcranial direct current stimulation (tDCS) with EFs training, can lead to improvements in language abilities in people with aphasia. Additionally, EFs training often results in specific effects on treated functions like working memory (near transfer effects) and untreated ones such as spoken sentence comprehension (far transfer effects). **Conclusions**: Despite the heterogeneity of the treatments and the small simple size of the studies analyzed, preliminary results are promising. Future research should further explore the effectiveness and specific contribution of EFs training to improving language functions in aphasia.

## 1. Introduction

Aphasia is an acquired language disorder following a stroke or traumatic brain injury [1] with a prevalence of 30–40% among individuals who have experienced a stroke or traumatic brain injury [2]. Depending on the cerebral network involved, aphasia can impact language domains including understanding spoken information, reading, writing, speaking, and other symbolic means of communication [3]. Aphasia affects approximately 4 million individuals worldwide post-stroke [4,5] and can lead to decreased quality of life, social participation, and overdependence on family members [6]. The literature suggests that up to 41% of people may present with aphasia during the acute phase post-stroke [7] and up to 34% may experience it as a chronic impairment [8]. The main cerebral structures involved in the language function are the left inferior frontal and posterior temporal regions, which are implicated in expressive and receptive abilities. The arcuate fasciculus, which connects these regions, is the focal point of the traditional model of language organization, known as the Broca–Wernicke–Lichtheim–Geschwind model [9,10]. This model is a classical framework for understanding language processing in the brain. It highlights two key areas: Broca’s area in the left frontal lobe, associated with speech production, and Wernicke’s area in the posterior superior temporal gyrus, linked to language comprehension. These regions are connected by the arcuate fasciculus, allowing the transfer of language information. According to the model, auditory inputs are processed in Wernicke’s area for comprehension, and Broca’s area produces speech by sending motor commands to the speech muscles. This model, while foundational, is now considered oversimplified as it omits many neural connections and subcortical regions involved in language processing [11,12]. However, recent neuroimaging studies have highlighted that language functions may include a broader network of cerebral regions involving bilateral cortical networks as well as subcortical circuits [13,14,15,16,17]. This perspective, known as neuronal multifunctionality, suggests that the brain’s complex neuronal circuits support both linguistic and non-linguistic information processing, resulting in mutual interactions between language and cognition [18,19]. Aphasia can result from damage to several brain regions, which overlap with regions that mediate working memory (WM) and executive functions (EFs) [20,21]. EFs are a wide range of high-level cognitive operations including planning, problem solving, criterion setting, multitasking, WM, cognitive control, inhibition, switching, and monitoring. These abilities are interconnected, yet independent [22,23].

Friedman and Miyake [24] suggest that WM, essential for the retention process of short-term linguistic information, is equally crucial for processing complex phrases. According to the literature, WM is considered as a part of EFs [25,26]. Moreover, EFs, especially interference control, contribute to sentence processing in aphasia [27]. Additionally, research [28,29,30] has demonstrated that WM and EFs deficits occurring in aphasia affect various language processes, such as lexical retrieval, sentence comprehension, and discourse production [27,31,32,33,34,35].

Cognitive deficits, such as attention and memory problems, frequently co-occur with other language difficulties [36,37]. In this regard, the literature shows that aphasiac patients perform worse on attention tasks compared to healthy adults, even when there are no language demands. These tasks include dividing attention [38,39], alternating attention [40], sustaining attention [41,42], and orienting attention [43].

Therapeutic strategies for aphasia encompass a wide range of approaches, which can be broadly categorized into restorative and compensatory treatments, based on their underlying principles and targeted outcomes. Restorative treatments aim to improve impaired language functions by targeting neuroplasticity through structured practice. Examples include traditional repetitive language exercises, enriched environments, and Constraint-Induced Aphasia Therapy. Enriched environments integrate sensory, social, and cognitive activities to promote neuroplasticity [44,45], while Constraint-Induced Aphasia Therapy emphasizes intensive verbal practice and minimizes compensatory behaviors to enhance linguistic performance [46,47]. System activation therapies, such as mirror neuron system activation, rely on action observation and imitation tasks to stimulate neural plasticity [44,45]. These therapies share some overlap with intensive practice methods but are distinguished by their focus on leveraging neural circuits for specific linguistic functions. Non-invasive neurostimulation treatments, including transcranial magnetic stimulation and transcranial direct current stimulation (tDCS), modulate cortical activity and can complement traditional speech and language therapy (SLT) by enhancing neural responsiveness [48].

Compensatory treatments, on the other hand, aim to develop alternative strategies for communication and often rely on EFs, such as planning, problem-solving, and WM. Examples include augmentative and alternative communication tools, which provide external aids for communication, and communication partner training, which focuses on facilitating interaction through strategies like simplified language and written cues [46,47,49]. Script training helps individuals practice predictable dialogues, while problem-solving therapy focuses on identifying and overcoming communication barriers. Telerehabilitation provides a flexible service delivery model that facilitates access to intensive therapy in home-based settings [50]. Unlike direct interventions targeting language functions, these approaches often require significant cognitive engagement and highlight the interplay between language and executive processing.

Research by Worrall et al. [46,47] offers a useful framework for categorizing aphasia treatments into restorative and compensatory approaches, helping to clarify their respective mechanisms and goals. However, the distinction between treatment approaches (e.g., traditional SLT) and service delivery models (e.g., telerehabilitation) must be carefully maintained to avoid conflating their purposes. Furthermore, while many of these treatments demonstrate efficacy, the specific contributions of WM and EFs to their success remain underexplored. For instance, errorless naming therapy, a restorative approach, has been shown to improve outcomes by minimizing errors during practice, a process that may be influenced by underlying EFs capacities like WM and planning [51]. Similarly, compensatory methods often depend on cognitive functions to enable patients to develop and utilize alternative communication strategies effectively. A personalized approach to aphasia rehabilitation considers individual patients’ cognitive profiles, stroke severity, and specific response to different therapies. This approach can help tailor cognitive exercises and neuromodulation techniques to better address the unique needs of each patient, optimizing recovery outcomes.

The link between EFs and language is supported by both theoretical and empirical evidence. The Hierarchical Competing Systems Model suggests that language facilitates executive control by aiding information retention in WM and enabling conscious reflection [52,53]. Developmental changes in EFs are influenced by self-initiated labeling strategies that enhance conscious reflection and override habitual responses. Empirical studies have further connected EFs to language development, showing that components like inhibition and WM play crucial roles in lexical, semantic, and syntactic processing. For instance, inhibition helps resolve ambiguities and avoid overgeneralization [54,55], while WM supports semantic processing and error detection [56,57].

The relationship between EFs and language development in children is particularly evident in those with Developmental Language Disorder (DLD) [58]. Children with Developmental Language Disorder exhibit significant deficits in EFs, including WM, inhibition, cognitive flexibility, planning, and fluency, all of which are critical for linguistic processing. These impairments, confirmed by behavioral and neuroimaging studies, often extend to non-verbal EFs tasks and are associated with anomalies in frontal brain regions [59,60]. A study on EFs training in children with Developmental Language Disorder highlighted its potential to enhance language development. The computer-based program, focused on visuospatial WM, inhibition, and cognitive flexibility, led to significant improvements in these areas, along with sustained attention and behavioral outcomes. These cognitive gains, observed both immediately and six months post-training, suggest that enhancing EFs may indirectly support language development by strengthening the underlying cognitive processes essential for linguistic tasks [61]. Some studies have revealed that EFs components, like WM and inhibition, predict language abilities in structural and pragmatic contexts [62]. Experimental tasks have demonstrated similar relationships between EFs and language across children with Autism Spectrum Disorder, Developmental Language Disorder, and typical development, with individual differences in WM predicting performance in grammatical and lexical tasks [63,64]. These findings emphasize the complex and interdependent relationship between EFs and language across various developmental conditions. While current research highlights the importance of EFs in aphasia rehabilitation, gaps remain in understanding how targeted EFs training could enhance language recovery. EFs like planning, problem-solving, and strategy switching are particularly relevant in compensatory treatments, as they facilitate the development of adaptive communication strategies [65]. Moreover, integrating WM training into aphasia therapy may yield cascade effects, improving both cognitive and linguistic functions. These gaps underscore the need for further research into the relationship between EFs and aphasia rehabilitation to develop more effective and targeted interventions. This systematic review aims to address these gaps by synthesizing existing evidence and identifying areas for future exploration. The primary research question guiding this review was: how does EFs training, either alone or combined with traditional aphasia therapies, impact language abilities in people with aphasia (PWA)?

## 2. Materials and Methods

A systematic review was conducted to investigate if EFs rehabilitation approaches can augment language performance in people with aphasia. This systematic review adhered to the Preferred Reporting Items for Systematic Reviews and Meta-Analyses (PRISMA) guidelines [66] and has been registered in the Prospective Register of Systematic Reviews (PROSPERO 2024) with the number CRD42024519087.

### 2.1. PICO Model

We employed the PICO (Population, Intervention, Comparison, and Outcome) model to shape our research question [67]. Our target population comprised adults (>18 years) affected by post-stroke aphasia. The intervention involved various EFs training programs, either alone or combined with traditional rehabilitation approaches. For the comparison, we focused on patients who underwent a traditional rehabilitation approach or who did not undergo any training. The outcomes of the study include several key areas. First, language performance was assessed through standardized tests, focusing on measures such as comprehension, repetition, and naming scores. Additionally, improvements in functional communication abilities were evaluated, particularly in terms of the use of alternative strategies to express oneself and actively engage in daily interactions. Lastly, changes in the application of executive functions during communicative tasks were examined, using qualitative observations or specific assessment tools to capture these dynamics. Our research examines how different EF training approaches can support language recovery in individuals with aphasia, and investigates which specific aspects of language abilities are most likely to improve as a result of these rehabilitation programs.

### 2.2. Search Strategy

A systematic search was conducted for all peer-reviewed articles, starting in February 2024 and covering publications up until that date using the following databases: Scopus, PubMed, Web of Science, and Embase. We considered WM to be a component of EF. The search combined the following terms in PubMed: (“executive function”[MeSH] OR “executive function”[All Fields] OR (“executive”[All Fields] AND “function”[All Fields])) AND (“education”[MeSH Subheading] OR “education”[MeSH] OR Education[All Fields] OR train*[All Fields] OR program*[All Fields] OR schooling[All Fields]) AND (aphasi*[All Fields]) AND (“rehabilitation”[MeSH] OR “rehabilitation”[MeSH Subheading] OR Rehabilita*[All Fields]).

All articles were reviewed based on titles, abstracts, and full texts by two investigators (L.C. and D.L.), who independently performed data collection to reduce the risk of bias. The researchers read the full-text articles deemed suitable for the study and in case of disagreement on the inclusion and exclusion criteria, the final decision was made by a third researcher (V.L.B). The list of articles was then refined for relevance, revised, and summarized, with the key topics identified from the summary based on the inclusion/exclusion criteria.

#### Inclusion and Exclusion Criteria

The inclusion criteria were: (i) adult patients with post-stroke aphasia; (ii) studies that implemented EFs training programs for adult patients with post-stroke aphasia; (iii) studies that reported both neuropsychological and linguistic outcome measures pre- and post-treatment; (iv) studies that explored the impact of EFs training on cognitive (executive functions) and linguistic outcomes in individuals with aphasia; (v) only articles written in English and (vi) published in a peer-reviewed journal; and (vii) the selected studies included Randomized Controlled Trials, cross-over, and quasi-experimental designs, including single-subject experimental designs with multiple baselines.

We excluded: (i) conference proceedings or commentaries; (ii) single case studies and reviews; and (iii) articles with a lack of assessment of EFs in people with aphasia.

### 2.3. Data Extraction and Analysis

Following the full-text selection, data were extracted from the included studies and reported in a table using Microsoft Excel (Version 2021). The extracted data included: study title, first author name, year of publication, study aims and design, sample size, type of participants, type of intervention and control, baseline performance, type of outcome and time-points for assessment, results, and key conclusions.

Moreover, the agreement between the two reviewers (L.C. and D.L.) was assessed using the kappa statistic. The kappa score, with an accepted threshold for substantial agreement set at >0.61, was interpreted to reflect substantial concordance between the reviewers. This criterion ensures a robust evaluation of the inter-rater reliability, emphasizing the achievement of a substantial level of agreement in the data extraction process.

### 2.4. Assessing the Quality of Included Studies—Risk of Bias

The risk of bias in controlled studies was independently assessed by D.L, L.C. and V.L.B using the revised Cochrane risk of bias (RoB 2) tool [68], which comprises five domains: (i) bias arising from the randomization process; (ii) bias due to deviations from the intended intervention; (iii) bias due to missing outcome data; (iv) bias in the measurement of the outcome; and (v) bias in the selection of the reported result. Regarding non randomized studies, we used the Cochrane tool for non-randomized controlled studies-of exposures (ROBINS-E) tool [69], which comprises seven domains: (i) bias due to confounding; (ii) bias arising from measurement of the exposure; (iii) bias in selection of participants into the study (or into the analysis); (iv) bias due to post-exposure interventions; (v) bias due to missing data; (vi) bias arising from measurement of the outcome; and (vii) bias in selection of reported result.

## 3. Results

**Figure 1:**
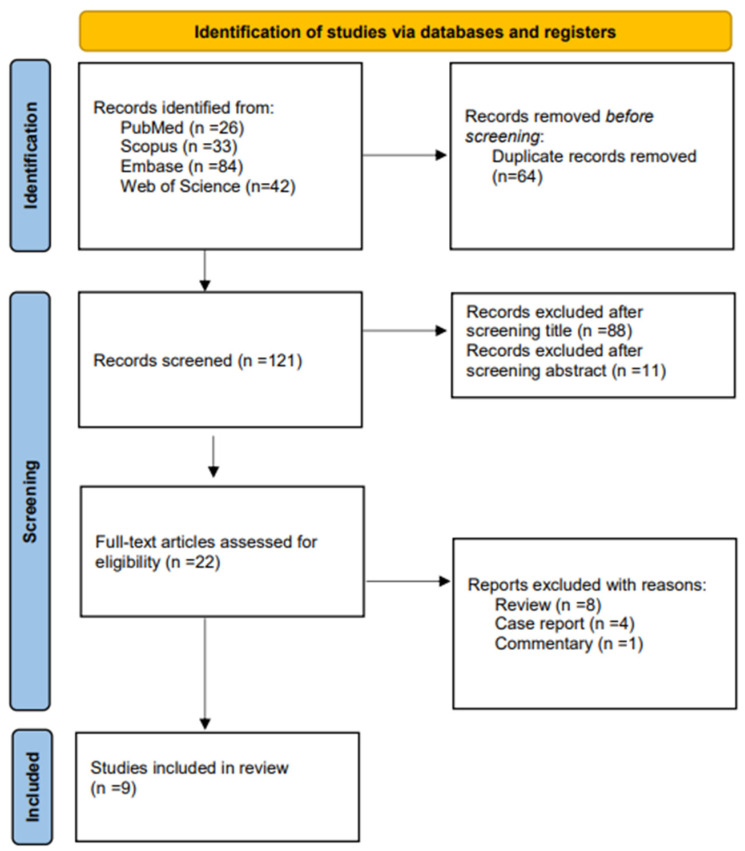
PRISMA flow chart.

The electronic searches generated 185 studies. Following the removal of duplicates, 88 studies were screened on title and 11 on abstract. The total of full-text articles that were independently assessed for eligibility by two authors (L.C., D.L.) was 22. Following full text selection, nine studies were included for analysis. The selection process is shown in Figure 1.

### 3.1. Overview of the Studies Selected

The study by Spitzer et al. [70] was conducted with native German speakers. It included 10 participants with left-hemisphere aphasia, who were recruited from local private practices and rehabilitation centers. All participants were in the late post-acute or chronic phase of aphasia (at least 6 months post-stroke), and with a mean age of 54 years. Therapy occurred over two weeks, with 2–3 sessions per day. A three-month follow-up was conducted to assess the sustainability of the effects. Fidelity was ensured through standardized sessions with predefined topics and structured materials for consistency across participants.

Bontemps et al. [71] recruited native French speakers with a mean age of 60.25 years (SD = 12.76) and an age range of 42 to 70 years. A total of four participants (two women, two men) were involved in the study, all of whom were in the chronic stage of recovery following a left hemispheric stroke, with at least 6 months post-stroke.

To assess the maintenance of treatment effects, a 4-week follow-up period was included in the study design. All therapy sessions were delivered by experienced speech-language pathologists, each with at least 10 years of experience working with PWA. The speech-language pathologists followed a standardized treatment protocol, with strict adherence to the materials and procedures outlined for the study, ensuring consistency across participants and sessions.

The study by Liu et al. (2022) [72] involved native Chinese speakers with participants’ ages ranging from 26 to 74 years (average 51.5 years). A total of 68 participants were split into an experimental group (n = 33) and a control group (n = 35). A 4-week follow-up assessed the effectiveness of the intervention and the maintenance of improvements. Therapy in the experimental group combined 30 min of traditional speech-language therapy (SLT) with 30 min of computer-assisted executive function training (CAET), while the control group received only SLT. The study ensured fidelity by following a standardized protocol under the supervision of experienced speech-language therapists.

In the study by Choinski et al. [73], conducted in Poland, 34 participants with aphasia following their first left-hemispheric stroke were included, with ages ranging from 30 to 82 years (mean 59). In total, 34 participants suffering from aphasia following their first left-hemispheric stroke were included in the study, comprising 22 males and 12 females. The study featured a 3-month follow-up assessment, though only 19 participants were available for post-treatment evaluation. Fidelity was ensured by administering the Dr. Neuronowski^®^ software under therapist supervision and following a standardized protocol.

Lee (2013) [74] conducted their study with English participants ranging from 57 to 83 years (mean age 71). Four participants with chronic aphasia were included, three with mild anomic aphasia and one with moderate conduction aphasia. The study involved follow-up probes after treatment, with a duration of 2 to 8 months depending on participant availability. Fidelity was maintained through the use of the Attention Process-training program delivered by a trained clinician or graduate student, with all sessions following a standardized protocol and participants providing self-reported motivation data.

The study by Nikravesh 2021 [75] was conducted with Persian participants aged 29 to 61 years (mean 49.55). A total of 25 participants with mild to moderate Broca’s aphasia were split into training (n = 13) and control (n = 12) groups. A 4-week follow-up period was included to assess the effectiveness of the WM training program. Regarding fidelity, while the study mentions that all treatment sessions were conducted by the researchers, it does not provide formal evaluation methods for fidelity. However, the treatments were consistently administered according to a structured protocol, and the groups were matched in terms of treatment dosage, age, gender, and the severity of aphasia.

Pisano et al. [76] conducted their study with native Italian speakers, with participants aged 50 to 72 years (mean 61.04). A total of 20 participants with severe chronic aphasia due to left ischemic stroke were included. A 4-week follow-up measured the persistence of treatment effects. Fidelity was ensured through the use of the standardized Cogniplus software and controlled tDCS in a blinded protocol.

Zakarias et al. 2016 [77] carried out their study with Hungarian participants aged 53 to 73 years (mean 54.6). Eight participants, divided into a training group (n = 3) and a control group (n = 5), participated in the study. The 4-week follow-up assessed treatment effects, with fidelity ensured through the standardized n-back task, and performance feedback was provided to ensure engagement.

The study by Zakarias et al. 2018 [78] was conducted with German participants aged 39 to 77 years (mean 56.9). Three participants, all with chronic aphasia post-stroke, were included. A 4–6 week follow-up assessed long-term effects. Fidelity was assured through trained clinicians and standardized administration of the n-back training system.

### 3.2. Key Findings from Included Studies

The studies reviewed (Table 1) illustrate the effectiveness of various EFs training programs in improving language abilities in PWA, organized by language modalities. In line with the research question of this review, we aim to investigate how EFs training, either alone or combined with traditional aphasia therapies, impacts language abilities in PWA, with a particular focus on different language modalities. A total of nine studies were analyzed in this review.

In terms of reading comprehension, auditory comprehension, and spoken sentence comprehension, five studies suggested that specific interventions can lead to significant improvements (Lee et al. [74]; Liu et al. [72]; Nikravesh et al. [75]; Zakariás et al. [77]; Zakariás et al. [78]). Regarding naming, four studies found improvements in this area following targeted interventions (Choinski et al. [73]; Pisano et al. [76], Nikravesh [75]; Bontemps et al. [71]). Finally, when it comes to sentence production, four studies demonstrated significant benefits (Liu et al. [72]; Bontemps et al. [71]; Zakariás et al. [78]; Spitzer et al. [70]). Table 2 summarizes which linguistic functions improved as a result of specific rehabilitation modalities.

Reading comprehension can be improved through specific interventions. A study has shown promise in enhancing reading comprehension [74]. Direct Attention Training (DAT) is an intervention designed to improve cognitive processes like WM, sustained attention, and executive control through repetitive, structured exercises. Combined with metacognitive components, DAT aims to stimulate the recovery of impaired neural networks and attentional processing, effectively enhancing reading comprehension in individuals with aphasia [74]. Two out of four participants demonstrated improvements in maze reading performance, the primary outcome measure. A participant’s performance increased from a baseline average of 14.2 correct items to 20.0 during treatment and further to 24.0 at an 8-month follow-up. Similarly, another participant improved from a baseline average of 14.6 correct items to 19.8 during treatment and to 22.4 at follow-up, indicating sustained gains over time [74]. However, a limitation of this study is the small sample size, since there were only 4 subjects.

Several interventions have shown promise in enhancing auditory comprehension, particularly as analyzed in two studies. CAET is a nonverbal cognitive intervention designed to improve executive functions such as WM, attention, and cognitive flexibility. This computer-based training uses interactive modules tailored to the individual’s cognitive abilities, offering exercises with adjustable difficulty levels to enhance cognitive and linguistic recovery in post-stroke aphasia patients. CAET combined with SLT results in a reduction in aphasia severity and improvements in spontaneous speech, speech repetition, and auditory comprehension [72]. The experimental group (CAET + SLT) showed greater improvements than the control group (SLT only). Spontaneous speech improved by 3.13 vs. 1.40 points (*p* = 0.009), auditory comprehension by 2.31 vs. 0.44 points (*p* < 0.001), and speech repetition by 2.34 vs. 0.58 points (*p* < 0.001). The Aphasia Quotient increased by 18.08 vs. 5.20 points (*p* < 0.001), demonstrating the added benefits of CAET. While the study assessed immediate post-treatment improvements, it did not investigate differences between various types of aphasia. Another study [75] reported that auditory comprehension, naming, and repetition could be improved by an intensive WM training program with exercises of increasing difficulty. Authors reported that significant gains were observed across multiple language domains in an experimental group compared to a control group. Spontaneous speech improved by an average of 2.8 points versus 1.4 points in the control group (*p* = 0.009), auditory comprehension increased by 2.15 points (*p* < 0.001), and speech repetition by 2.01 points (*p* < 0.001). The Aphasia Quotient also showed a significant increase, with a 16.15-point gain in the experimental group compared to a 5.2-point improvement in the control group (*p* < 0.001). These findings highlight the efficacy of WM and EFs training in enhancing key language abilities [75]. However, long-term benefits remain uncertain due to the absence of follow-up assessments.

For sentence production, certain software tools have shown effectiveness. Dr. Neuronowski^®^, a computer software method, focuses on different abilities such as WM and EFs, particularly temporal information processing. The program consists of 31 therapeutic games across nine modules, focusing on tasks such as temporal information processing in the millisecond range, sequencing abilities, verbal and non-verbal memory, and auditory perception. It could produce positive effects on verbal short-term WM, as well as in linguistic abilities, particularly sentence comprehension (e.g., an increase from M = 33.69 to M = 37.85, *p* = 0.002), grammar comprehension, fluency (e.g., an increase from M = 10.56 to M = 13.56, *p* = 0.009), naming, and phoneme discrimination (e.g., an increase from M = 20.78 to M = 22.44) [73]. Nevertheless, the only study analyzed faced significant limitations due to an unequal number of participants in the pre- and post-assessments versus the follow-up. Some participants were unable to attend the follow-up due to either new medical complications such as recurrent strokes or seizures, or restrictions imposed by COVID-19 lockdowns, impacting the long-term assessment of language recovery.

Spoken sentence comprehension in PWA could be improved by a computerized training approach utilizing an n-back task targeting WM, updating, and interference control, as demonstrated in two studies. The n-back task is a cognitive exercise designed to enhance WM and EFs, including interference control. Participants monitor a sequence of stimuli (e.g., letters, numbers, or sounds) and indicate when the current stimulus matches one present “n” steps earlier in the sequence. The inclusion of “lures”—stimuli resembling target items but not fulfilling the n-back condition—adds complexity and demands enhanced interference control [77]. Participants showed large to very large effect sizes on the Test for Reception of Grammar, with t-values of 4.070 (*p* = 0.007) and 6.035 (*p* = 0.001), indicating substantial gains in sentence comprehension [77]. However, the study involved a very small number of participants, limiting the generalizability of the findings. Further, the lack of a robust control group makes it difficult to definitively attribute improvements to the intervention alone, as opposed to recovery over time or placebo effects. Similarly, another study [78], involving the same two n-back tasks (one with pictures, one with spoken words), showed improvements in spoken sentence comprehension, functional communication, and everyday memory and ameliorations maintained at a 6-week follow-up. In terms of Correct Information Units, significant increases were observed both in the number of words produced (U = 17, *p* < 0.01) and in the number of Correct Information Units (U = 19, *p* < 0.05). Improvements in communicative ability and naming have also been observed.

The Cognitive Flexibility in Aphasia Therapy (CFAT) is a novel intervention that combines cognitive flexibility training with conventional aphasia therapy to enhance patients’ communication strategies in dynamic social interactions [70]. By focusing on key aspects such as responding to topic changes, addressing misunderstandings, and employing nonverbal communication strategies, CFAT aims to improve verbal language skills, communicative abilities, and verbal cognitive flexibility. This, in turn, enhances patients’ capacity to effectively participate in conversations, aligning with the primary goal of aphasia treatment: facilitating meaningful social interaction and communication [79]. Performance in verbal cognitive flexibility tasks showed large effect sizes (Cohen’s d ≥ 0.9), indicating substantial gains. Naming performance, as assessed by the Screening of Naming as well as the Bielefelder Wortfindungsscreening test, improved notably, with participants showing a significant increase in word retrieval accuracy. Verbal fluency, particularly in semantic category shifts, exhibited significant gains (*p* < 0.05). Real-life communicative abilities measured by the Szenario Test improved moderately, with participants demonstrating enhanced multimodal communication skills. CFAT was more effective than conventional Everyday Language Therapy, yielding significant improvements in both trained topics (*p* < 0.001) and untrained topics (*p* = 0.004), highlighting the generalization of skills beyond targeted areas.

Semantic Feature Analysis is a therapy approach aimed at improving naming skills by strengthening the semantic network associated with words. It involves activating related semantic features of a target word, such as its category, use, or location, to enhance word retrieval.

The study by Bontemps et al. [71] combined EFs training with Semantic Feature Analysis, improving naming skills and discourse efficacy in PWA with these effects being sustained over time. Participants demonstrated significant improvements in discourse informativeness, as measured by the percentage of Correct Information Units, increasing from baseline (48.7%) to post-treatment (59.25%) and follow-up (62.7%) (*p* = 0.002). However, the small sample size (four participants) limits the generalizability of the findings.

Moreover, non-invasive brain stimulation techniques, particularly ten days of tDCS over the right dorsolateral prefrontal cortex (DLPFC), combined with EFs training resulted in persistent amelioration across multiple domains in people with severe aphasia. These included cognitive abilities such as planning, selective attention, and spatial WM, as well as linguistic functions. Functional communication scores increased substantially, from ~20 at baseline (T0) to ~50 post-treatment (T10). Oral noun naming improved markedly, from 5 correct responses at T0 to 30 at T10 (*p* < 0.001). Additionally, auditory and written sentence comprehension demonstrated statistically significant gains (*p* < 0.001). These findings highlight the potential of combined tDCS and EFs training to enhance both cognitive and linguistic outcomes in people with severe aphasia [76]. For personalized medicine applications, the studies reviewed incorporated varying degrees of individualized therapy based on patients’ cognitive and linguistic profiles. This customization of rehabilitation strategies aligns with the principles of personalized medicine, ensuring treatments are adapted to the unique characteristics of each individual.

### 3.3. Risk of Bias

**Figure 2:**
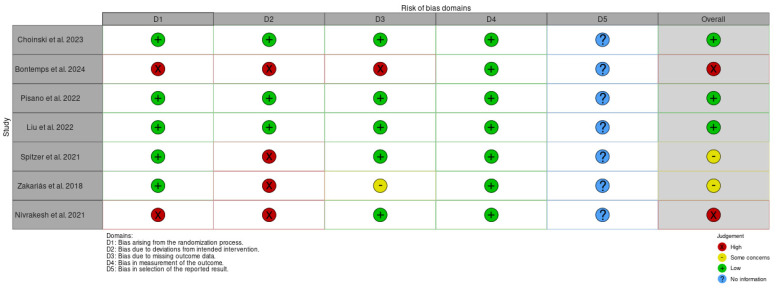
Shows the Risk of Bias (RoB 2) of studies regarding the main effective EFs training for language deficits in aphasia [70,71,72,73,75,76,78].

**Figure 3:**
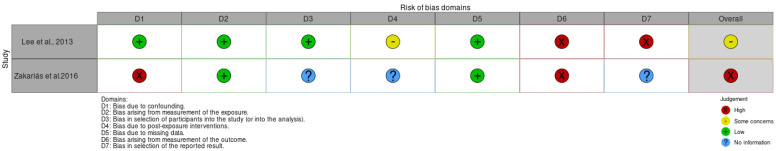
Shows the Risk of Bias (ROBINS-E) of studies regarding the main effective EFs training for language deficits in aphasia [74,77].

The Cochrane risk of bias (RoB 2) tool and the Risk Of Bias In Non-randomized Studies-of Exposures (ROBINS-E) tool were used to assess the risk of bias of the articles included in this review. Figure 2 and Figure 3 show the summary of the risk of bias assessment, while the graphs depict the distribution of bias concerns across the studies included. Out of the total studies assessed, three studies [72,73,76] showed a low risk of bias and robust methodologies.

In contrast, other studies exhibited a high risk of bias and weak methodologies. In particular, two studies [60,66] showed bias arising from the randomization process and four studies [60,63,64,66] showed bias due to deviations from the intended intervention. Only one study [66] displayed bias due to missing outcome data.

Additionally, another study [58] raised some concerns about bias due to post-exposure interventions and a high risk of bias arising from measurement of outcome and in the selection of reported results.

Moreover, only one study [77] displayed a high risk of bias due to confounding, and bias arising from the measurement of the outcome.

## 4. Discussion

The aim of this review is to describe EFs training methods, either alone or in combination with other traditional approaches, that may improve language abilities in aphasia.

EFs, crucial for complex cognitive processing, involve high-level cognitive skills including planning, problem-solving, cognitive flexibility, and memory manipulation, as well as attentional control processes [26]. In this review, EFs training explicitly includes tasks aimed at enhancing attention and WM, given their pivotal roles in supporting cognitive control and language recovery mechanisms. Research shows that improvements in these areas can significantly influence language rehabilitation outcomes in aphasia [80].

The body of evidence reviewed consistently demonstrates that EFs training, both in isolation and when combined with other therapeutic approaches, can enhance various aspects of language function in PWA. Many studies employed a combination of EFs training with traditional language therapies, suggesting that a holistic approach to aphasia rehabilitation is widely recognized as beneficial. The common outcomes across these studies included improvements in naming, verbal fluency, and reading comprehension. These are indicative of the transfer effects of enhanced EFs to broad language processing abilities.

EFs training can stimulate neural adaptation and plasticity in brain regions that support both EFs and language. Evidence suggests that EFs training may activate neural plasticity, particularly in regions overlapping with language networks, such as the left prefrontal cortex [81]. By better managing cognitive resources through trained EFs, individuals may enhance their abilities in language tasks, particularly under conditions of high cognitive demand or complexity [80]. Lastly, improvements in inhibitory control, a key aspect of EFs, allow individuals to better suppress irrelevant information, leading to clearer and more focused language use [82]. This suggests that by strengthening cognitive control mechanisms, EFs training can lead to improvements in language tasks, particularly those that require higher cognitive demands, such as sentence comprehension and verbal fluency. These results underscore the relevance of EFs training in clinical settings, where cognitive impairments often complicate language rehabilitation.

Difficulties in adapting to alternative communication strategies, such as gesturing or pointing to communication boards, have been observed in individuals with EFs impairments [29,83]. This inability to switch between methods can be attributed to deficits in cognitive flexibility, a core component of EFs. The findings are particularly relevant in the context of aphasia rehabilitation, as they underscore the critical role of EFs in supporting not only linguistic recovery but also the broader adaptability needed for effective communication. For instance, EFs training may enable individuals with post-stroke aphasia to better manage communicative demands by strengthening their ability to shift strategies in dynamic social interactions. These insights align with evidence suggesting that EFs training not only improves linguistic outcomes but also enhances the cognitive flexibility required to engage in diverse and meaningful communication scenarios [84]. Thus, integrating EFs training into aphasia rehabilitation could provide a dual benefit: promoting language recovery and fostering adaptive communication skills in everyday contexts [9].

It is well known that subjects with aphasia have attention disorders [85] that exacerbate language difficulties. Preliminary findings [86] showed that the ecological language-specific attention treatment is effective in the language and attention deficits in PWA, particularly in patients with a moderate degree of attention disorder.

DAT and Attention Process-training were highlighted in the results as effective interventions for improving attention and WM in PWA. The observed benefits of DAT, particularly in reading comprehension, align with the allocation theory of attention in aphasia [87], which posits that enhanced attentional control and WM can lead to measurable improvements in language modalities such as reading comprehension [74]. For example, DAT’s structured, repetitive drills were shown to improve reading maze performance in participants, with sustained gains over an eight-month follow-up period. These results suggest that DAT may help restore impaired neural circuits by promoting attention and WM recovery, thereby facilitating improved comprehension [74]. Additionally, CAET can complement traditional language rehabilitation, resulting in beneficial outcomes for cognitive function and language recovery, particularly auditory comprehension [72]. As auditory comprehension improves, the prognosis also ameliorates. This suggests the potential inclusion of CAET in linguistic rehabilitation. The improvement may be attributed to training-induced brain plasticity [88] or domain-specific network effects on language functions [87].

Clinically, it is clear that EFs training can complement traditional aphasia therapies by targeting underlying cognitive deficits that may be hindering language recovery. For instance, interventions like DAT and CAET, which enhance WM and attention, can be used alongside standard language therapies to promote more comprehensive language improvements. Clinicians should consider integrating EFs training into their practice, particularly for patients with cognitive impairments that impede language recovery. According to previous studies [89,90], the WM training program is efficacy in improving not only WM functions but also language domains in aphasia [75]. Improvements in naming and verbal fluency after WM training have been widely documented in the literature [75,78,91]. These enhancements may result from far-transfer effects to domains different from those trained, sharing the same mental processes [92,93]. However, there are controversial results in the literature regarding the effectiveness of WM treatment in improving sentence comprehension. Some studies have reported positive effects [94], while others have shown no ameliorations [95].

The benefits of WM training extended not only to WM tasks but also to unpracticed WM tasks, as well as spoken sentence comprehension, functional communication, and everyday memory. These results align with theories suggesting that WM improvement allows PWA to employ alternative approaches and strategies to address language comprehension deficits [96,97,98,99].

Regarding CFAT, two strengths are noted: language improvements generalized to other communication contexts, and its individualized approach allowing therapists to use personalized materials, like personal photos of PWA, to increase treatment adherence [70]. However, clinicians should consider that CFAT focuses only on cognitive flexibility. Integrating other rehabilitation approaches focusing on WM and other EFs crucial for daily communication may be beneficial [80].

Moreover, a key clinical implication is the need for individualized therapy. Given the variability in cognitive and linguistic profiles among PWA, clinicians should tailor EFs training programs to each patient’s specific needs, focusing on the areas that will most benefit their communication and cognitive abilities [100,101]. For example, using techniques like Semantic Feature Analysis in combination with EFs training can further enhance naming and discourse abilities [102,103].

A notable imbalance in sex distribution was observed in several studies, such as those conducted by Nikravesh et al. [75], Spitzer et al. [70], Liu et al. [72], and Choinski et al. [73], where a greater number of male patients were recruited. This bias could potentially influence the generalizability of the findings, as sex differences might affect the outcomes of EFs training due to biological and sociocultural factors. Additionally, the variability in aphasia types among study participants presents another layer of complexity. The studies included in our review encompassed a range of aphasia severities and types, from mild anomic to severe global aphasia. This diversity, while beneficial in showcasing the broad applicability of EFs training, also complicates the interpretation of efficacy across different aphasia profiles.

Although the current literature underscores the potential benefits of EF training, there is a lack of large-scale, longitudinal studies that could provide more definitive conclusions about the long-term benefits and the sustainability of post-intervention improvements. Additionally, incorporating neuroimaging and other advanced assessment tools may provide deeper insights into the neural mechanisms underlying observed improvements. This could help in tailoring more effective and targeted interventions.

Some studies within this review demonstrated strong methodological practices and low risk of bias, enhancing the credibility of their findings [72,73,76]. However, others [71,75] exhibited significant weaknesses, including biases in randomization, intervention adherence, outcome measurement, and handling of confounding variables, highlighting the need for more rigorous study designs in future research. We have also considered the impact of publication bias, recognizing that studies with non-significant results are less likely to be published, potentially skewing the evidence base towards positive findings. Addressing these methodological issues is crucial for advancing the evidence base on the effectiveness of EFs training in aphasia rehabilitation, ensuring that future findings are both reliable and generalizable.

Clinically, integrating EFs training with traditional therapies and leveraging technology can significantly enhance patient outcomes, paving the way for more personalized and effective treatment protocols. Clinicians are encouraged to consider EFs training as a complementary therapy, particularly for patients whose aphasia is complicated by cognitive deficits.

There are a paucity of studies investigating the efficacy of non-invasive brain stimulation on language rehabilitation. Particularly, neuronavigated excitatory transcranial magnetic stimulation applied to the Broca’s area improved language abilities in people with chronic post-stroke aphasia. Functional magnetic resonance imaging results showed that this amelioration correlated with a stronger language lateralization to the left hemisphere [104]. An interesting study [105] has provided a preliminary indication about the efficacy of the combination of computerized WM training and intermittent Theta Burst Stimulation of the left dorsolateral prefrontal cortex, ameliorating language, memory, fluid intelligence, and quality of life, though not all benefits persisted at the three-month follow-up, indicating the need for further investigation.

One of the most promising techniques for the rehabilitation of cognitive skills, including language, is tDCS. The Memory-Unification-Control model shows that the left dorsolateral prefrontal cortex, where cognitive control and WM are located, is necessary for language comprehension and production [106,107]. In this regard, Pisano et al. [76] reported that tDCS over the dorsolateral prefrontal cortex combined with EFs training improves functional communication in people with severe aphasia. This result is in line with a past study showing an improvement in verbal fluency as well as in speed of naming high frequency words after anodal tDCS over the dorsolateral prefrontal cortex [108]. However, the study by Pisano and colleagues [76] does not isolate the effects of tDCS alone or EFs training alone. Thus, it remains challenging to discern which component is more critical for the observed improvements or whether their synergy is necessary for the best outcomes.

The interventions analyzed suggest the potential for personalized rehabilitation approaches that integrate cognitive training with neurostimulation techniques, a key tenet of personalized medicine. The improvement observed in cognitive and linguistic domains highlights the importance of targeting specific brain regions and cognitive functions for individualized care.

In the studies reviewed, various training programs were proposed to enhance EFs which were measured before and after treatment. The tests administered, such as the Paced auditory serial addition test, categorization working memory span, Corsi Block-Tapping Test Backwards, and the Wisconsin Card-Sorting Test, varied based on the specific EFs to be measured (WM, problem solving, cognitive control).

Previous studies have highlighted the challenge of assessing EFs in PWA due to the overlap between EFs assessment tools and language functions. For instance, while certain EFs tests, like the design generation subtest of the Cognitive Linguistic Quick Test, are entirely non-linguistic, others involve some language processing. Assessments like the Wisconsin Card Sorting Test and various Tower tasks encompass multiple EF skills, making it difficult to directly correlate performance with specific EF abilities of interest. On the contrary, Cognitive Linguistic Quick Test subtests are best suited for screening isolated skills. Therefore, clinicians should carefully consider which assessment procedure is most appropriate.

### 4.1. Study Strengths and Limitations

This systematic review has several strengths. While EFs encompass various skills, the studies under consideration particularly emphasize the efficacy of targeted WM training, which has yet been extensively documented in the literature [109]. WM involves retaining information online while performing other cognitive tasks and it is closely integrated with language [110]. However, this review also indicates that other functions, such as interference control and cognitive flexibility, could be trained to improve language skills, providing suggestions for clinical practice.

The main limitations related to the literature we reviewed should be mentioned. First, there is a limited number of papers meeting the inclusion criteria, with heterogeneous methodologies and interventions, limiting robust evidence. The variability of interventions and trained functions, such as WM or cognitive flexibility, consequently determines positive effects on different linguistic domains.

Additionally, most studies report preliminary results, precluding generalization. Moreover, the studies selected predominantly recruited small samples, with a predominance of females, and involved individuals with left hemisphere damage and various forms and severity of aphasia. These several limitations underscore the need for future studies.

### 4.2. Future Directions

Despite the variability in treatment procedures and participants’ characteristics, we found positive outcomes following EFs training in PWA. Future research should quantify the specific contribution of EFs training to the improvement of language functions in aphasia. While promising, the generalizability of EFs training programs to language performance highlights the need for further research and controlled implementation to explore their potential inclusion in aphasia rehabilitation programs with careful measurement of outcomes.

## 5. Conclusions

In conclusion, this review highlights that EFs training can lead to improvements in the linguistic skills of PWA. EFs are not a unitary construct but comprise many different subcomponents and processes which can be selectively preserved. Targeting these processes more precisely may lead to the augmentation of language performance. A further methodological improvement for future studies could be increasing the sample size and standardizing a rehabilitation procedure. Despite the heterogeneous methodology and interventions preventing us from drawing robust evidence, this review may help guide the design of future studies as well as emphasize the importance of tailored approaches to address the specific needs of individuals with aphasia. Personalized approaches to aphasia rehabilitation, including tailored cognitive training and the use of non-invasive neuromodulation, show promise for improving language outcomes. Future research should explore how genetic, cognitive, and functional profiles can guide treatment decisions to maximize individual recovery.

## Figures and Tables

**Table 1:** Main characteristics of the included studies.

Authors	Type of Study	Sample Size	Age (M ± SD)	Aphasia Severity and Type	Lesion Site	Time Since Onset	Mother Language of Patients	Rehabilitation Approach	Treatment Dosage	Language Test	EFs Test	Main Findings
Choinski et al., 2023 [73]	RCT	34 (22 male)	59 ± 13	Moderate to severe Aphasia	Left hemispheric stroke	lesion age: Me = 32 weeks; min–max: 5–195 weeks	Polish	Dr. Neuronowski^®^ training targeting WM, inhibitory control, planning and cognitive flexibility, selective and sustained attention	24 sessions, 45 min each, three times a week over 8 weeks	Token test, Grammar-Sentence Comprehension Test, Vocabulary-Word Comprehension Test, Phoneme Hearing Test	TOL; Verbal Memory Test Backwards, Corsi Block-Tapping Test Backwards	The experimental training improves TIP, WM as well as sentence and grammar comprehension (Token Test, *p* = 0.002), fluency (*p* = 0.009) and naming performance (*p* = 0.045)
Bontemps et al., 2024 [71]	Concurrent MBD across participant	4 (2 female)	60.25 ± 12.76	From very severity to moderate Chronic aphasia (two fluent, two non-fluent).	Left hemispheric stroke	Mean time post-stroke was 54.75 months (SD: 70.48, range 11–160)	French	EFs training and SFA targeting cognitive flexibility, inhibition, WM, planning	12 sessions, 45 min each, three times a week over 4 weeks, with 15–20 min of EF training and 25–30 min of SFA therapy per session	Picture naming task from the BETL. Subtest of BDAE and WAB	MCST; verbal fluency task	The combined EF training and SFA therapy improved naming accuracy (treated items: +35%, untreated items: +20%), discourse efficiency (+25% words/min), and informativeness (+30% CIUs), with gains maintained over time in 75% of participants (3/4).
Pisano et al., 2022 [76]	Randomized cross-over design	20 (10 female)	61.04 ± 7.02	Sever non fluent aphasia	Left ischemic stroke	6 months prior to the investigation	Italian	tDCS over DLPFC and EFs training particularly alertness, selective attention, visuo-spatial memory and planning	10 sessions, 1 h each, five days a week over 2 weeks	EDL II; Token test; BADA, CADL-2	CADL-2; attention visual search; short term memory Corsi test; non verbal Smirni subtes; TOL	tDCS over DLPFC combined with EFs training improve communication (CADL-2: +25%, *p* ≤ 0.001), noun and verb naming (*p* ≤ 0.001), and written and auditory sentence comprehension (*p* ≤ 0.001), with effects maintained at 1-month follow-up
Lee et al., 2013 [74]	A single-subject, multiple baseline design	4 (3 female)	71 years	Chronic aphasia. 3 people with mild anomic aphasia and 1 with moderate conduction aphasia (2 mild, 1 moderate)	Left ischemic CVA	From 18 to 79 months (M = 43 months)	English	APT-3 program includes WM, executive control and attention tasks	30–45 min per session, administered four times a week for a duration of 8 weeks	GORT-4; RCBA-2;	TEA; CPT-II	APT-3 training, combining direct attention training with metacognitive facilitation, improved maze reading comprehension (effect sizes: d = 2.46, 2.58), sustained attention, and executive control in 50% of participants, with gains maintained up to 8 months post-intervention.
Liu et al., 2022 [72]	Single blind randomized trial	33 (10 female)	51.5 ± 15.1	Mild to moderate aphasia 4: anomic 12: Broca 5: Wernicke 8: Global 4: non classified	Ischemic stroke: 29 people Hemorrhagic stroke: 4 people	Days after onset: 28.3 (range, 15–73)	Chinese	Traditional speech, SLT and CAET targeting WM, inhibitory control, cognitive flexibility, problem solving and abstract reasoning	4 weeks, 6 days per week: experimental group received 30 min of CAET and 30 min of SLT daily; control group received 30 min of SLT twice daily.	WAB	VFT, the Proverbs Test, TOL, TMT, SCWT	CAET combined with SLT significantly improved auditory comprehension (η^2^ = 0.43, *p* < 0.001), aphasia quotient (AQ, η^2^ = 0.26, *p* < 0.001), and cognitive function (e.g., TMT-B: η^2^ = 0.12, *p* = 0.005), demonstrating enhanced language and executive skills
Zakariás et al., 2016 [77]	Combined pre/post-test case control design	3 (1 female)	54.6± 15.58	Moderate to severe aphasia. 2 transcortical motor aphasia and 1 anomic aphasia	Left hemisphere infarct	Post onset (month): 108.8 (100.61)	Hungarian	A modified n-back task targeting WM, interference control, cognitive flexibility	13 sessions, 20 min each, 3–4 times per week over 4 weeks with an adaptive n-back task.	WAB, two language tasks	Two n-back tasks	WM and EFs training improved interference control and updating, leading to significant gains in spoken sentence comprehension (TROG-H: +9%, *p* = 0.008) and visual n-back task performance (*p* = 0.045)
Spitzer et al., 2021 [70]	Cross-over	10 (4 female)	54 years	Moderate aphasia. 3 Amnestic; 2 Broca, 1 global, 1 Wernicke, 3 fluent non-classifiable	Left-hemisphere aphasia	>6 months post-onset	German	CAFT (targeting cognitive flexibility, problem solving and adaptability, WM, inhibitory control, nonverbal communication skills) and conventional aphasia therapy	20 sessions over 2 weeks, 2–3 sessions per day, using a cross-over design with CFAT and ELT interventions	BIWOS; Szenario-Test; AAT; ELT	WCST-64; RWT; CFA-Screening	CFAT significantly improved language skills, verbal cognitive flexibility (semantic fluency: d = 0.91), and communicative ability (Szenario-Test: d = 0.47), with gains extending to untrained topics and maintained at 3-month follow-up.
Zakariás et al., 2018 [78].	Multiple-baseline (with control) experimental design	3 female	39, 77, 51 years	Moderate to severe. 1 Broca; 2 unclassified	Left hemisphere stroke	6, 25, 15 years	German	Visual n-back task with pictures and an auditory n-back task with spoken words. The tasks target WM, interference control, cognitive flexibility, sustained attention	16 sessions, 25–35 min each, 3–4 times per week over 4–5 weeks, with pre/post-tests and follow-up after 4–6 weeks	ANELT; Sätze verstehen; Token test; TROG-D; EMQ	N-back with letters; Running span	WM training using n-back tasks improved sentence comprehension (TROG-D: +12%, *p* = 0.01) and functional communication (ANELT scores: +15%, *p* = 0.02), showing transfer effects from cognitive to linguistic domains
Nivrakesh et al., 2021 [75]	Quasi-experimental study	13 (2 female)	48.77± 10.02	Mild to moderate Broca’aphasia.	Left hemisphere damage	25.23 (mean SD 17.96)	Persian	WM training program	15 sessions, 60 min each, conducted twice a week with a 10-min break per session	PAB; BDAE; P-WAB-1	n-Back test; PASAT; CWMS; DMST;	training improved performance in trained tasks (CWMS: η^2^ = 0.83, *p* < 0.001) and untrained WM tasks (n-back: η^2^ = 0.61, *p* < 0.001), with far transfer effects on language abilities (AQ: +6.69, η^2^ = 0.71, *p* < 0.001).

Legend = TOL: Tower of London; EDL II: esame del linguaggio II; BADA: Batteria per l’analisi dei deficit afasici; CADL 2: Communication Activities of the Daily Living; SLT: language therapy; WAB: Western Aphasia Battery; VFT: verbal fluency test; SCWT: Stroop Color and Word Test, TMT: Trail Making Test; AQ: aphasia quotient; CFAT: Cognitive Flexibility in Aphasia Therapy; CFA-Screening: Cognitive Flexibility in Aphasia Screening; ELT: Everyday Language Therapy; PAB: Persian Aphasia Battery; BDAE: Boston Diagnostic Aphasia Examination; PASAT: paced auditory serial addition test; CWMS: categorization working memory span; DMST: forward and backward digit memory span tests; P-WAB-1: Bedside Version of Persian; CVA: cerebrovascular accident; TIP: Temporal Information Processing; MCST: Modified Card Sorting Test; SFA: semantic feature analysis; GORT-4: Gray Oral Reading Test-Fourth Edition; RCBA-2: Reading Comprehension Battery for Aphasia; TEA: test of everyday attention; CPT-II: Conners’s Continuous Performance test-II; RWT: Regensburger Wortfluessigkeits-Test; WCST: Wisconsin Card-Sorting Test-64; BIWOS: Bielefelder Wortfindungsscreening für leichte Aphasien; AAT: Aachener Aphasie Test; ANELT: Amsterdam-Nijmegen Everyday Language Test; TROG-D: Test for Reception of Grammar-Version 2; EMQ: Everyday memory questionnaire; APT-3: Attention Process-training.

**Table 2:** Improvements in language skills categorized by modality.

Study	Intervention	Language Modalities Improved
Lee et al. [74]	Direct attention training	Reading comprehension
Liu et al. [72]	Computer-assisted executive control training (CAET) combined with SLT	Auditory comprehension, spontaneous speech, speech repetition
Nivrakesh et al. [75]	Intensive WM training	Auditory comprehension, naming, repetition
Choinski et al. [73]	Dr. Neuronowski^®^ software	Sentence comprehension, grammar, fluency, naming, phoneme discrimination
Zakariás et al. [77]	Computerized training (n-back task for WM, updating, interference control)	Spoken sentence comprehension
Zakariás et al. [78]	n-back tasks (pictures, spoken words)	Spoken sentence comprehension, functional communication
Spitzer et al. [70]	Cognitive Flexibility in Aphasia Therapy (CFAT)	Verbal language skills, communicative ability
Bontemps et al. [71]	EFs training combined with semantic feature analysis (SFA)	Naming skills, discourse efficacy
Pisano et al. [76]	Transcranial direct current stimulation (tDCS) with EFs training	Functional communication, naming

## Data Availability

Not applicable.
